# Misleading hepatitis B testing in the setting of intravenous immunoglobulin

**DOI:** 10.12688/f1000research.2-249.v1

**Published:** 2013-11-18

**Authors:** Christelle M Ilboudo, Erin M Guest, Angela M Ferguson, Uttam Garg, Mary Anne Jackson

**Affiliations:** 1 Children’s Mercy Hospitals and Clinics, Division of Infectious Diseases and University of Missouri-Kansas City School of Medicine, Kansas City, MO, 64108, USA; 2 Children’s Mercy Hospitals and Clinics, Department of Pediatrics, Division of Hematology Oncology and University of Missouri-Kansas City School of Medicine, Kansas City, MO, 64108, USA; 3 Children’s Mercy Hospital and Clinics, Department of Pathology and Laboratory Medicine, Division of Laboratory Medicine and University of Missouri-Kansas City School of Medicine, Kansas City, MO, 64108, USA

## Abstract

Intravenous immunoglobulin (IVIG) is commonly used for a wide range of diagnoses, by multiple pediatric subspecialists. We report two cases of hepatitis B screening results post IVIG infusion, where positive anti-Hepatitis B core antigen serology tests indicated possible occult hepatitis infection, leading to a delay in care. However, serial antibody testing showed results consistent with the passive transfer of antibodies.

## Introduction

Intravenous immunoglobulin (IVIG) is a blood product prepared by pooling plasma from 3000–10,000 healthy blood donors. Adverse events are reported in 1–15% of treated patients, and most clinicians are aware of common events such as infusion reactions, and more serious reactions that follow IVIG infusion including renal dysfunction (a US Boxed warning), thrombotic events, anti-globulin hemolysis, and aseptic meningitis syndrome ^1^.

Since IVIG is a passive antibody transfer, it can result in transiently positive anti-viral serology tests. We report two cases where screening hepatitis B testing resulted in an unusual pattern of immunoglobulin positivity after IVIG therapy prompting additional laboratory testing and delayed treatment in one of the children. Based on the known half-life of IVIG products of 21 days, we used serial testing of sera to confirm degradation of antibody over time. Clinicians should be aware that passive transfer of antibodies is expected and serologic screening should be performed pre-treatment if IVIG therapy is anticipated.

## Case reports

### Patient 1

A 6 year old African-American female presented with several weeks of bruising and epistaxis. A diagnosis of Evans syndrome and systemic lupus erythematosus was confirmed and she received IVIG in the form of Gamunex™ on days 1, 9, 10 and 18 at doses of 1 gram/kg each time. On day 19, hepatitis screening (Ortho-Clinical Diagnostics VITROS 5600 system Raritan, NJ) was performed in anticipation of possible initiation of rituximab therapy. The results were positive for anti-Hepatitis B core antigen (anti-HBc) and negative for Hepatitis B surface antigen (HBsAg) ( Table 1). She had received 3 doses of Hepatitis B vaccine as an infant, had no prior transfusions and her liver function testing was normal. She was born in the US, had never traveled outside of the US and had no other exposures or risk factors for hepatitis B. Specific hepatitis B testing consisting of HBsAg, anti-HBc which is the total immunoglobulin level against the core antigen, anti-Hepatitis B core antigen specific IgM (IgM anti-HBc), hepatitis B e antigen (HBeAg) and antibody to HBeAg (anti-HBe) was performed on day 23 post-IVIG. The testing was positive for anti-HBc and anti-Hepatitis B e-antigen (anti-HBe), but negative for IgM anti-HBc. The IgM anti-HBc is important to determine whether the total anti-HBc result is consistent with an acute infection or reflective of a chronic or resolved infection ( Table 2). The combination of positive anti-HBc and anti-HBe raised the concern of acute, resolved or chronic hepatitis B infection. As a result of the misleading results, the initiation of rituximab therapy was delayed, until further evaluation was completed showing antibody degradation consistent with the passive transfer of antibodies ( Table 1). The same hepatitis B specific testing was repeated a month later, since the half-life of IVIG is 21 days. The repeated testing was negative for both anti-HBc and anti-HBe which confirmed the suspicion that her initial positive screen and specific hepatitis B testing were the results of IVIG infusion. Interestingly, her anti-HBs decreased as well over time, further evidence of the IVIG being the source of the immunoglobulins detected.

**Table 1.** Patient 1 and 2 hepatitis B diagnostic tests.

	Patient 1			Patient 2			
Testing ^≠^	Day 19	Day 23	Day 57	Pre-IVIG	Day 7	Day 8	Day 105
Hepatitis B surface antigen (HBsAg)	Negative	Negative	-	Negative	Negative	Negative	Negative
Antibody to HBsAg (anti-HBs)*	-	>1000	187.00	-	-	404.00	110.00
Hepatitis e antigen (HBeAg)	-	-	Negative	-	-	Negative	Negative
Antibody to HBeAg (anti-HBe)	-	Reactive	Negative	-	-	Negative	-
Total antibody to Hepatitis B core antigen (anti-HBc)	Reactive	Reactive	Negative	Negative	Reactive	Reactive	Negative
IgM antibody to Hepatitis B core antigen (IgM anti-HBc)	-	Negative	-	-	-	Negative	Negative

^≠^Number of days post IVIG infusion

*Values are in milliInternational Units/ml: > 12 is adequate immunity either from previous infection or vaccines

-Testing not done

**Table 2.** Diagnostic Tests for HBV*.

Factors being tested	HBV antigen or antibody	Interpretation
HBsAg	Hepatitis B surface antigen	Detection of acutely or chronically infected people
Anti-HBs	Antibody to HBsAg	Identification of people who have resolved infections; determination of immunity after immunization
HBeAg	Hepatitis e antigen	Identification of infected people at increased risk of transmitting HBV
Anti-HBe	Antibody to HBeAg	Identification of infected people with lower risk of transmitting HBV
Anti-HBc (total)	Antibody to HBcAg	Identification of people with acute, resolved or chronic HBV infection (not present after immunization)
IgM anti-HBc	IgM antibody to HBcAg	Identification of people with acute or recent HBV infections (including HBsAg-negative people during the “window” phase of infection)

*Adapted from: Committee on Infectious Diseases *et al.* Red Book Online 369–390 ^7^. HBV: hepatitis B virus

### Patient 2

An 11 year old Caucasian female with high risk pre B-cell lymphoblastic leukemia failed to enter into remission with standard chemotherapy regimens and was enrolled in the National Cancer Institute study, Anti-CD19 White Blood Cells for Children and Young Adults with B Cell Leukemia or Lymphoma (NCI identifier NCT01593696). In preparation for the cellular therapy, she was given IVIG in the form of Gamunex™ at 0.5 gram/kg once on day 1. Hepatitis B screening (Ortho-Clinical Diagnostics VITROS 5600 system Raritan, NJ) performed in anticipation of bone marrow transplantation, 7 days following IVIG therapy showed a positive anti-HBc with a negative HBsAg, raising the possibility of acute, resolved or chronic hepatitis B infection ( Table 1). She had normal liver function at the time of the reactive results. This child was born in the US and had received 3 doses of hepatitis B vaccine as an infant. She had not traveled outside of the US and had no other risk factors for Hepatitis B infection. She had previously received multiple packed red blood cells and platelet transfusions. Additional data confirmed that hepatitis screening had been conducted at another facility, 2 months prior to the IVIG administration and that test result was negative. Table 1 shows serial results of antibody testing consistent with passive antibody degradation. The serial testing consisted of specific hepatitis B testing, using the same laboratory system, on day 8 post-IVIG and 3 months later which revealed a negative IgM anti-HBc. Interestingly, her anti-HBs decreased as well over time as further evidence of IVIG being the source of the immunoglobulins detected. In this case, given her negative prior screening test and her negative IgM anti-HBc, her transplantation was delayed until the results of the testing on day 8 post–IVIG was available. She did receive her transplantation a month prior to the last hepatitis B specific testing.

## Discussion

We report two instances where an unusual pattern of hepatitis B positivity related to IVIG infusion complicated the care of children with an underlying autoimmune or oncologic diagnosis.

A variety of serologic tests are available to confirm hepatitis B infection, and typically more than one marker is present in acute or chronic infection. The pattern of hepatitis B seropositivity noted in our two patients, where anti-HBc was present, while less common, is compatible with occult hepatitis infection.

The association of anti-HBc positivity with certain IVIG products was previously noted by Arnold *et al.* during a rituximab study of patients with refractory immune-mediated thrombocytopenia ^2^. Pre-treatment hepatitis screening was conducted on 24 study patients because of the known risk for hepatitis B reactivation following rituximab. Anti-HBc positivity was found in 45% of the cohort and the investigators noted that IVIG use was common in their patient group. They were able to show seroreversion over time in 10/11 of their IVIG-treated patients, consistent with degradation of passively transferred antibody. They tested five immune globulin preparations and confirmed anti-HBc presence in three, including Gamunex™, the preparation received by our patients. Benton *et al.* additionally described a patient being considered for rituximab therapy whose care was delayed secondary to misleading hepatitis B testing post IVIG infusion ^3^. A false positive enzyme immunoassay and a *Treponema pallidum* haemagglutinin assay have also been reported following IVIG infusion ^4, 5^. Positive human T-lymphotropic virus (HTLV) testing following IVIG infusion for chemotherapy-induced polyneuropathy has also been reported in a leukemic patient resulting in a delay in his cord blood transplantation ^6^. In all the above cases, repeated testing 4–8 weeks post IVIG revealed lower or negative antibody titers.

There are 11 immune globulin preparations available in the US and Canada, and specific diagnostic indications, FDA approved ages, dosing recommendations, infusion rates, IgA composition and routes of administration vary by product ( Table 3). Based on the number of diagnoses for which IVIG is used, a wide range of subspecialists are involved in prescribing IVIG and education regarding passive antibody transfer therefore needs to be targeted to clinicians in those subspecialties such as neurology, immunology, hepatology, hematology and rheumatology as well as general practitioners.

**Table 3.** Immune globulin products available in the US.

Product brand name	IgA content mcg/mL	FDA approved diagnosis	Hepatitis core antibody evaluated/found ^2^	Approved pediatric age/comments
Bivigam™ 10%	≤200	Primary immunodeficiency	Not done	≥ 6 years; FDA approved, but not available
Carimune ^®^NF ^2^ 3 and 12%	Trace	Primary immunodeficiency, Acute/chronic idiopathic thrombocytopenic purpura (ITP)	Not done	Pediatric patients; intravenous (IV)
Fleebogamma ^®^DIF 5 and 10%	<50–100	Primary immunodeficiency	Not done	IV
GamaSTAN™ S/D	Not applicable	Passive immunoprophylaxis, prophylaxis in immunodeficiency	Not done	Pediatric patients for immunoprophylaxis measles, varicella, hepatitis A; IV
Gammagard S/D ^®^ 5% and 10%	≤2.2–4.4	Primary immunodeficiency, B-cell Chronic Lymphocytic Leukemia (CLL), acute/chronic ITP, Kawasaki disease	Not done	IV and subcutaneous
Gammagard Liquid 10%	37	Primary immunodeficiency	YES	Pediatric patients 2–16 years; IV
Gammaked™ 10%	46	Primary immunodeficiency, Acute ITP, chronic inflammatory demyelinating polyneuropathy	Not done	Pediatric patients; IV
Gammaplex ^®^ 5%	<10	Primary immunodeficiency	Not done	Pediatric patients; IV
Gamunex ^®^-C	46	Primary immunodeficiency, acute/chronic ITP, chronic inflammatory demyelinating polyneuropathy	YES	Pediatric patients; IV
Hizentra ^®^	≤50	Primary immunodeficiency	Not done	Pediatric patients ≥ 2 years; Subcutaneous only
Octagam ^®^	≤200	Primary immunodeficiency	Not done	Pediatric patients 6–16 years; IV
Privigen ^®^	≤25	Primary immunodeficiency, Acute ITP	YES	Pediatric patients ≥ 3 years; IV

Our cases serve to remind clinicians of the potential for passive antibody transfer with IVIG products and to define the potential clinical confusion that can arise in situations where infectious serologic screening is not performed pre-infusion. In all situations where IVIG is prescribed, the patient’s underlying diagnosis, clinical indication, the product brand and dose, lot number and specific infusion instructions should be explicitly outlined and all baseline serologic specimens, when indicated, obtained before infusion of the product.

## Consent

Written informed consent for publication of their clinical details was obtained from the parent/guardians of the patients.
