# Supplementary material for: Misleading hepatitis B testing in the setting of intravenous immunoglobulin
Source: F1000Res. 2013 Nov 18;2:249. [Version 1] doi: 10.12688/f1000research.2-249.v1 (PMC4103488; doi:10.12688/f1000research.2-249.v1)
Supplement: Peer review history

# Peer review: Referee response for version 1

**DOI:** 10.5256/f1000research.2799.r4223

**Published:** 2014-03-24

**Authors:** Melvin Berger

**Affiliations:** 1 Immunology Research & Development, CSL Behring, King of Prussia, PA, USA

I cannot say this better than the authors have: *'Our cases serve to remind clinicians of the potential for passive antibody transfer with IVIG products and to define the potential clinical confusion that can arise in situations where infectious serologic screening is not performed pre-infusion'*. It is important to realize that this includes HBcAb, since it is not generally recognized that antibodies to hepatitis B antigens other than HBSAg (HBsAb) are present in US IgG products, as shown in Table 3. It is also important to recognize that passive transfer of antibodies also occurs with subcutaneous IgG treatment. The gold standard for identifying blood borne infection is detection of viral genomes in the patient's blood. Nucleic acid tests such as PCR are done to rule out the presence of infectious agents during the processing of plasma for products like IVIG, and are available in many hospital and commercial reference laboratories. Prompt confirmation of the absence of detectable hepatitis B virus DNA in the patient's blood could obviate the need to wait for catabolism and dropping of the titer of passively transferred antibody, and avoided the delays experienced by the patients described in this paper.

I have no conflicts of interest regarding this paper. For the purposes of complete disclosure, I am a salaried employee of CSL Behring, LLC, a manufacturer of fractionated plasma products like IVIG, and a stock-holder.

I have read this submission. I believe that I have an appropriate level of expertise to confirm that it is of an acceptable scientific standard.

# Peer review: Referee response for version 1

**DOI:** 10.5256/f1000research.2799.r2483

**Published:** 2013-12-12

**Authors:** Adlette Inati

**Affiliations:** 1 Division of Pediatric Hematology and Oncology, Children's Center for Cancer and Blood Diseases, Rafik Hariri University Hospital, Beirut, Lebanon

Intravenous immunoglobulin (IVIG) infusions are widely used for various medical indications. Since these products are pooled from thousands of healthy blood donors, it is possible to have passive antibody transfer of certain donor-related infections to the IVIG recipient. In this report Ilboudo *et al.* present two cases where IVIG infusions led to false positive transient hepatitis B titers and delayed the management of two critical patients. Previous reports have also indicated such an observation. Accordingly, this article serves as a reminder of possible positivity of hepatitis titers post IVIG that would resolve in few weeks. It also underscores the importance of screening for infections including hepatitis B pre IVIG infusion. This article is well written and lists the IVIG products available in the US with significant comments that can be used as a reference.

I have read this submission. I believe that I have an appropriate level of expertise to confirm that it is of an acceptable scientific standard.
